# Application of Magnetic Particles for Fast Determination of Immunoreactive Fraction of ^68^Ga-Labelled VHH Antibodies to PD-L1

**DOI:** 10.17691/stm2023.15.3.03

**Published:** 2023-05-28

**Authors:** K.O. Avrov, S.V. Shatik, V.V. Zaitsev, R.I. Al-Shehadat, O.A. Shashkova, L.A. Terekhina, I.S. Malakhov, M.P. Samoylovich

**Affiliations:** Senior Researcher, Laboratory of Hybridome Technology; A.M. Granov Russian Research Center of Radiology and Surgical Technologies of the Ministry of Health of the Russian Federation, 70 Leningradskaya St., Saint Petersburg, Pesochniy Settlement, 197758, Russia;; Head of the Department of Cyclotron-Produced Radiopharmaceuticals; A.M. Granov Russian Research Center of Radiology and Surgical Technologies of the Ministry of Health of the Russian Federation, 70 Leningradskaya St., Saint Petersburg, Pesochniy Settlement, 197758, Russia;; Head of the Group for Radiopharmaceuticals Synthesis; Leading Technologist, Department of Cyclotron-Produced Radiopharmaceuticals; A.M. Granov Russian Research Center of Radiology and Surgical Technologies of the Ministry of Health of the Russian Federation, 70 Leningradskaya St., Saint Petersburg, Pesochniy Settlement, 197758, Russia;; General Director; Innova plus LLC, 13 Kalinina St., Lit. A, Office 18-N, Saint Petersburg, 197198, Russia;; Senior Researcher, Laboratory of Hybridome Technology; A.M. Granov Russian Research Center of Radiology and Surgical Technologies of the Ministry of Health of the Russian Federation, 70 Leningradskaya St., Saint Petersburg, Pesochniy Settlement, 197758, Russia;; Researcher, Laboratory of Hybridome Technology; A.M. Granov Russian Research Center of Radiology and Surgical Technologies of the Ministry of Health of the Russian Federation, 70 Leningradskaya St., Saint Petersburg, Pesochniy Settlement, 197758, Russia;; Senior Researcher, Laboratory of Hybridome technology; A.M. Granov Russian Research Center of Radiology and Surgical Technologies of the Ministry of Health of the Russian Federation, 70 Leningradskaya St., Saint Petersburg, Pesochniy Settlement, 197758, Russia;; Chief Researcher^1, 3^; Head of the Laboratory of Hybridome technology; A.M. Granov Russian Research Center of Radiology and Surgical Technologies of the Ministry of Health of the Russian Federation, 70 Leningradskaya St., Saint Petersburg, Pesochniy Settlement, 197758, Russia;

**Keywords:** magnetic particles, immunoreactive fraction, radioimmunoconjugate, VHH antibodies

## Abstract

Quantification of the immunoreactive fraction (IRF) of radioactive isotope-labeled antibodies or their fragments is necessary to assess the specific activity of radiopharmaceuticals. Traditionally, cells expressing the target molecules on their surface are used to determine IRF, but such analysis is time-consuming and has difficulties with standardization.

**The aim of the study** was to develop a fast and reliable method for quantitative determination of IRF by ^68^Ga-labeled VHH antibodies to PD-L1 based on the use of magnetic particles coated with antigen molecules.

**Materials and Methods:**

Commercially available magnetic particles coated with protein A have been used in our study. The antigen conjugated with the Fc fragment (PD-L1-Fc) was immobilized on the particles. The IRF value of ^68^Ga radionuclide-labeled nanobodies (VHH) against PD-L1 (^68^Ga-VHH-PD-L1) was determined using magnetic particles coated with antigen molecules and cells expressing the antigen on their surface. When VHH antibodies were conjugated to ^68^Ga radionuclide, protein molecules were modified using bifunctional chelating agents: tetraazacyclododecanetetraacetic acid (DOTA) or deferoxamine (DFO). The magnitude of IRF was defined as the ratio of radioactivity specifically bound to particles or cells to the total radioactivity added to the sample.

**Results:**

The specificity of the ^68^Ga-VHH-PD-L1 radioimmunoconjugate binding to the antigen-coated magnetic particles has been proved. Some special aspects, which should be taken into consideration when using this method, have been established. The comparison of the IRF estimates using the antigen-expressing cells and magnetic particles has not revealed any significant differences in the results obtained in our study. Nevertheless, the presented method based on magnetic particles with immobilized antigen molecules requires only 15 min to determine the radioimmunoconjugate IRF, which is of fundamental importance for the routine assessment of the specificity of radiopharmaceuticals containing short-lived isotopes.

## Introduction

Radioimmunoconjugates based on antibodies or their fragments are designed for targeted delivery of radionuclides to the cells having appropriate antigens on their surface in order to diagnose and treat tumor diseases. However, radionuclide labeling may reduce antigen-binding capacity of macromolecules [1]. Therefore, production of such pharmaceuticals requires the assessment of their specific activity. For this purpose, the value of immunoreactive fraction (IRF) is determined, i.e. a relation of radioactivity of the labeled molecules capable of specific binding to the antigen to the total radioactivity of the labeled radioimmunoconjugate molecules. Traditionally, radioimmunoconjugates are incubated with the tumor cells expressing the target molecules on their surface [1-6]. This approach is connected with a number of limitations and drawbacks. Besides, the level of antigen expression by the cells may be insufficient for the determination of radioimmunoconjugate IRF [7-10]. Besides, the cell-based study is time-consuming which is critical when the preparation contains a short-lived radionuclide. There are difficulties with standardization of cell application for IRF estimation: the cells may express different isoforms of target molecules; the level of antigen expression in these cells changes over time in the process of their passaging [10-12].

By the present time, few works have been published in which the specific activity of monoclonal antibody-based radioimmunoconjugates was studied using magnetic particles coated with immobilized antigen molecules [10, 13–15]. We were the first to define the conditions of using this method for evaluation of the specific activity of radionuclide-labeled VHH antibodies (nanoantibodies). In the work presented here, magnetic particles were applied for estimating IRF of ^68^Ga-labeled preparations of nanoantibodies against PD-L1 protein developed jointly by the A.M. Granov Russian Research Center of Radiology and Surgical Technologies (Russia) and Innova plus LLC (Russia).

A ligand of the programmed cell death receptor PD-1, PD-L1, is one of the proteins of the immune checkpoints. This transmembrane protein plays an important role in inhibition of the T-cell-mediated immune response [16, 17]. PD-L1 is expressed on the antigen-presenting and tumor cells [16, 18], which is connected with a poor prognosis and increased mortality from various cancer diseases [19, 20]. PD-L1 is used as a biomarker in tumor treatment including melanoma, lung cancer, breast cancer, renal cancer, and other types of cancer [18, 21]. In recent time, a lot of papers have been published, in which application of radioimmunoconjugates with antibodies against PD-L1 for cancer therapy and diagnosis is studied including the works with ^68^Ga-labeled VHH antibodies [22-25]. The authors used the method by Lindmo [1] to determine the IRF of these pharmaceuticals employed in positron emission tomography. For this purpose, tumor gene-modified human cells A375-hPD-L1 with an increased level of expression of the PD-L1 recombinant protein were used [22]. However, ^68^Ga radionuclide has a 68-min half-life period, which is too short for determining the IRF of these preparations by the cells-based technique. For the clinical application of radioimmunoconjugates containing ^68^Ga, a faster method of estimating their specific activity is required.

**The aim of the study** was to develop a fast and reliable method for quantitative determination of IRF by ^68^Ga-labeled VHH antibodies to PD-L1 based on magnetic particles coated with antigen molecules.

## Materials and Methods

### Labeling of VHH antibodies with ^68^Ga radionuclide

 When conjugating VHH antibodies (Innova plus LLC, Russia) with ^68^Ga radionuclide, the protein molecules were modified using bifunctional chelating agents (BCAs): tetraazacyclododecantetraacetic acid (DOTA, an isothiocyanate derivative) or deferoxamine (DFO, an ethyl squarate derivative). These chelators were attached to the protein molecules via the primary amine groups by incubation of 1–2 mg of protein in 50 mM of borate or sodium carbonate buffer (pН 8.5–8.8) with a 10-fold BCA excess for 17 h at room temperature. The modified proteins were separated by gel filtration chromatography in 50-mM ammonium acetate buffer solution (pН 7.5), the desired fraction was concentrated using ultrafiltration. To label the protein with ^68^Ga, 100 μg of the modified protein was placed into the test tube, 50-μl solution of the [^68^Ga]GaCl_3_ in 0.1 M of HCl, obtained by elution of gallium-68 generator (Cyclotron CJSC, Russia) and 150 μl of 50-mM ammonium acetate buffer solution (pН 75) were added. When labeling DFO-modified VHH antibodies, the reaction mixture was incubated at 37°C for 15 min in the presence of air and stirred in the thermoshaker (1000 rpm). When labeling DOTA-modified VHH antibodies, the reaction mixture was incubated under the same conditions except for the temperature, which was increased to 60–70ºC. Incorporation of the radionuclides into the protein molecules exceeded 95%.

### Determination of ^68^Ga-VHH-PD-L1 radioimmuno-conjugate IRF using magnetic particles

 To determine IRF of the ^68^Ga-VHH-PD-L1 radioimmunoconjugate, commercially available magnetic particles of 2.4–3.4 μm in diameter coated with protein A (category No.161-4013; Bio-Rad, USA) were used. A magnetic stand (NPF Helicon LLC, Russia) was employed for the work with the particles. The centrifuge tube with particles was removed from the magnetic stand before the addition of a new portion of the solution and installed back before its elimination.

For immobilization of the antigen conjugated to Fc fragment (PD-L1-Fc) on the magnetic particles, 200 μl of magnetic particle suspension was placed in the 1.5-ml centrifuge tube secured in the magnetic stand, the solution was removed. Then, the particles were washed in 1 ml of the solution containing 50 mM of NaCl, 50 mM of Tris HCl, 0.05%-Tween-20 (pH 7.6) — TBST. The particles were incubated for 15 min with 1 ml of PD-L1-Fc solution in TBST (10 μg/ml). Incubation was performed on the rotary mixer at 15 rpm. For determination of nonspecific binding, particles not coated with antigen were prepared. In this case, TBST was added to the particles instead of PD-L1-Fc solution. Further, the particles were washed in 1 ml of the TBST solution containing 1% of bovine serum albumin (BSA) and incubated in this solution for 5 min, the solution was then removed and 200 μl of TBST-BSA was added.

In the process of IRF determination, 50 μl of ^68^Ga-VHH-PD-L1 radioimmunoconjugate solution from the series of dilutions in TBST with different concentrations and volumetric activity of radioimmunoconjugate was added to 10 μl of the particle suspension. The last was added both to the particles coated with antigen and to those without antigen coating (to define nonspecific binding). After the incubation, the samples were washed three times with TBST for 15 min, then the value of sample radioactivity was measured using a Triathler radiometer designed for measuring radionuclide activity (Hidex Oy, Finland). The radioactivity value of the ^68^Ga-VHH-PD-L1 molecules specifically bound to the magnetic particles (*B*) coated with the antigen molecules was determined by the formula:

B=A−N,

where *A* is the value of radioactivity bound to the PD-L1-coated particles; *N* is the value of radioactivity bound to the magnetic particles not coated with the antigen molecules. The IRF values were calculated using the formula:

IRF=B/T⋅100%,

where *T* is the value of the total radioactivity added to the sample.

To determine the IRF, a plot of the *B*/*T* ratio versus the concentration of the radioimmunoconjugate in the sample was built. IRF was defined by the sample having the maximum *B*/*T* value.

Nonspecific binding was also determined by adding unlabeled VHH-PD-L1 molecules to the sample with antigen-coated particles (a blocking method). Unlabeled VHH antibodies were added to the sample 1 min before the addition of radioimmunoconjugate at the concentration 200 times higher than the concentration of the latter. After the incubation, the samples were washed to remove unbound radioactivity as described above.

All measured values of radioactivity were recounted for the time of measuring the first sample (zero time point) by the formula:

A0=At⋅eλt,

where *A*_0_ is sample radioactivity at the zero point, *A_t_* is sample radioactivity measured *t* minutes after the first sample measurement was started, λ is the decay constant of the ^68^Ga isotope (0.010237).

### Determining IRF of ^68^Ga-VHH-PD-L1 radioimmunoconjugate using CT26-PD-L1 cells in vitro

 Numerical values of the radioimmunoconjugate IRF were defined according to the method based on the use of the constant amount of the radionuclide-labeled antibodies and variable amount of antigen (cells) [1]. CT26-PD-L1 cells were obtained in our laboratory by genetic engineering from the mouse adenocarcinoma cell line CT26 and expressed on average 1.5 million human PD-L1 molecules on their surface. Cells taken from the vial surfaces or cells, which were *ex tempore* unfrozen after cryopreservation, were used. In both variants, the cells were suspended in the phosphate-buffered saline solution (PBS) containing 1% of BCA and 0.05% of sodium azide. The number of cells was defined using a Z1 Series Coulter counter (Beckman Coulter, USA) and their viability was evaluated using a trypan blue solution (BioloT, Russia). All procedures were carried out at 4°C. The cell suspensions with progressively decreasing cell concentration were dispensed into Eppendorf test tubes, and then an equal amount of the labeled VHH antibodies was introduced into all test tubes. The test tubes with maximum number of cells contained 4.0 million, with minimum number — 0.0625 million of cells. The cell samples were incubated for 1 h in the solutions of the labeled antibodies suspending them periodically with the help of the Vortex mixer (VELP Scientifica, Italy) to prevent cell sedimentation. The samples were then washed 3 times in PBS by centrifugation to remove unbound antibodies. Sample radioactivity was determined using the Triathler radionuclide activity radiometer (Hidex Oy, Finland). To define nonspecific bonding, the samples were preincubated with similar unlabeled antibodies taken in 100-fold excess compared to the concentration of the radionuclide-labeled antibodies. The IRF percentage was determined by the sample, in which the maximum value of specific binding of all labeled VHH antibodies with the target molecules expressed on the surface of the cells has been reached. Further increase of the cell content in the sample at the same concentration did not lead to the increase of specific binding; it spoke of the fact that all intact labeled VHHs have specifically bound to the cells.

### Statistical data processing

 All results were obtained in at least three repetitions. The experimental data were processed using Statistica 10.0 software package. The data are presented as M±SD, where M is a mean, SD is standard deviation. Statistical differences were assessed by a pairwise comparison of independent samples using the Mann–Whitney test; differences with a significance level of p<0.05 were considered statistically significant.

## Results and Discussion

The objectives of the study were as follows: to evaluate the possibility of using magnetic particles for determining IRF of the ^68^Ga-VHH-PD-L1 radioimmunoconjugate, to define optimal analysis conditions for this method, and to compare it with traditional cell-based incubation.

**Figure 1.**
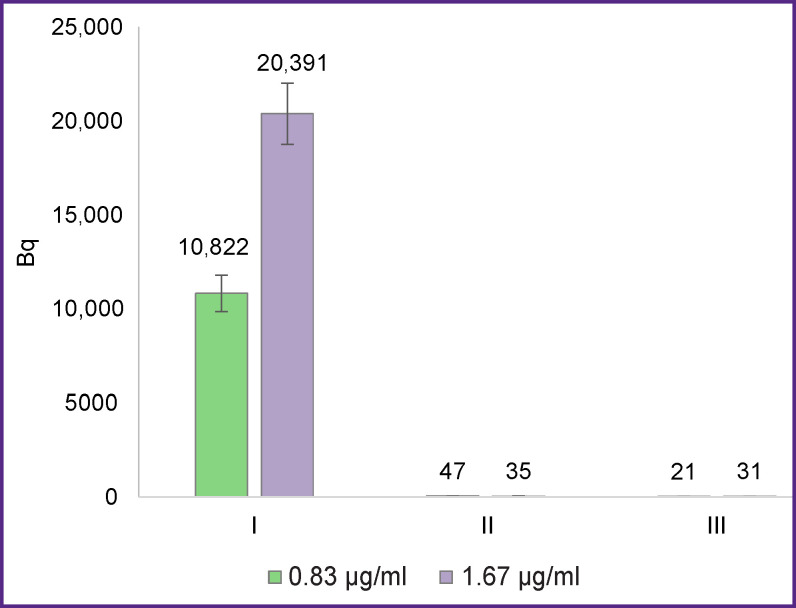
Determination of specific and nonspecific binding of the ^68^Ga-VHH-PD-L1 radioimmunoconjugate to magnetic particles: I — binding of radioimmunoconjugate to antigen-coated magnetic particles; II — binding of radioimmunoconjugate to magnetic particles coated with antigen molecules in the presence of a high concentration of unlabeled VHHs; III — binding of radioimmunoconjugate to magnetic particles not coated with antigen molecules. Figures in the graph show the value of the sample radioactivity. Concentration of the radioimmunoconjugate is indicated in the legend

There are two approaches to testing the specificity of binding of a radioimmunoconjugate to magnetic particles: blocking of radioimmunoconjugate binding to antigen-coated particles by the excess of unlabeled VHHs and binding of radioimmunoconjugate to the particles not coated with the antigen molecules (Figure 1).

Data presented in Figure 1 demonstrate that binding of the ^68^Ga-VHH-PD-L1 radioimmunoconjugate to the magnetic particles in the sample was effectively blocked by a high concentration of unlabeled VHHs against PD-L1. Nonspecific binding was similarly low in the absence of antigen molecules on the surface of the particles. This proves the specificity of binding radioimmunoconjugate to the antigen-bearing particles and also justifies the application of the particles without antigen for measuring nonspecific binding. Notably that in all experiments presented in this paper, the level of nonspecific binding did not exceed 1% of the total level.

**Figure 2.**
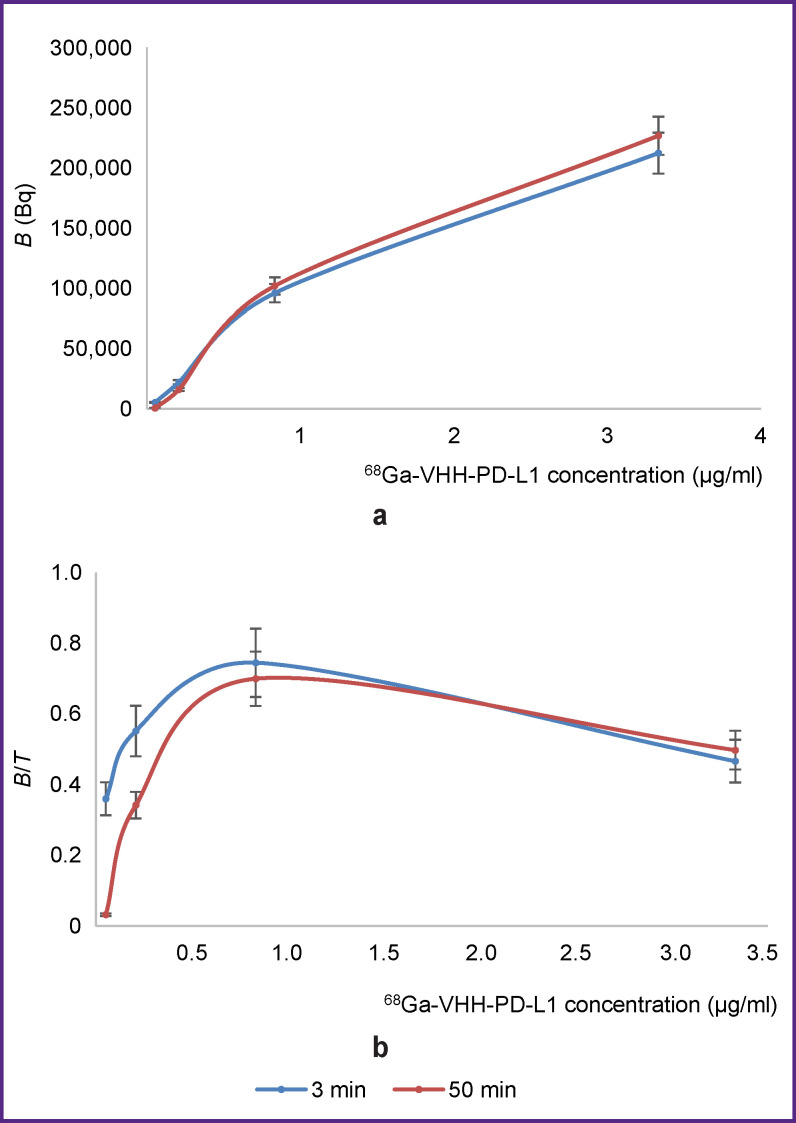
The effect of incubation time on specific binding of the ^68^Ga-VHH-PD-L1 radioimmunoconjugate to the antigen-coated magnetic particles (a) and on the value of *B*/*T* ratio (b) Here and further: *В* is the radioactivity value of the ^68^Ga-VHH-PD-L1 molecules specifically bound to magnetic particles coated with antigen molecules; *T* is the value of total radioactivity added to the sample. The time of incubation of radioimmunoconjugate with magnetic particles is indicated in the legend

The results of determining the dependence of the measured IRF value of the ^68^Ga-VHH-PD-L1 radioimmunoconjugate on the time of its incubation with magnetic particles are shown in Figure 2.

The time of incubation of the ^68^Ga-VHH-PD-L radioimmunoconjugate with antigen-coated magnetic particles did not essentially influence the value of specific binding (Figure 2 (a)). The *В*/*T* value did not also depend on the time of incubation (Figure 2 (b)). Thus, three-minute incubation is sufficient for radiopharmaceutical testing, which is critically important for the application of short-lived isotopes. When particles are incubated with a radioimmunoconjugate for 3 min, the entire analysis, including radioactivity measurement in the samples, takes no more than 15 min. It makes it possible to quickly evaluate specific activity of radioimmunoconjugates containing short-lived isotopes such as ^68^Ga having a 68-min half-life. It should be noted that determination of radioimmunoconjugate IRF by a traditional way of using cells requires more than 2 h. Further application of radioimmunoconjugates containing short-lived isotopes as radiopharmaceuticals becomes impossible.

At high values of ^68^Ga-VHH-PD-L1 concentration (3.33 μg/ml) in the sample, the value of *B*/*T* ratio reduced compared to its maximum magnitude obtained at 0.83 μg/ml concentration. It may be explained by the excess of the labeled VHH antibodies relative to the antigen immobilized on the particles. Here, it is important to note that incubation of the antigen-coated particles with radioimmunoconjugate at the concentration below 0.83 μg/ml also resulted in the decreased *B*/*T* value. The results obtained demonstrate that in order to measure the maximum *B*/*T* value, it is necessary to select experimentally the ratio of radioimmunoconjugate concentration to that of the magnetic particles in the sample. It will enable to quantitatively assess the radioimmunoconjugate IRF.

**Figure 3.**
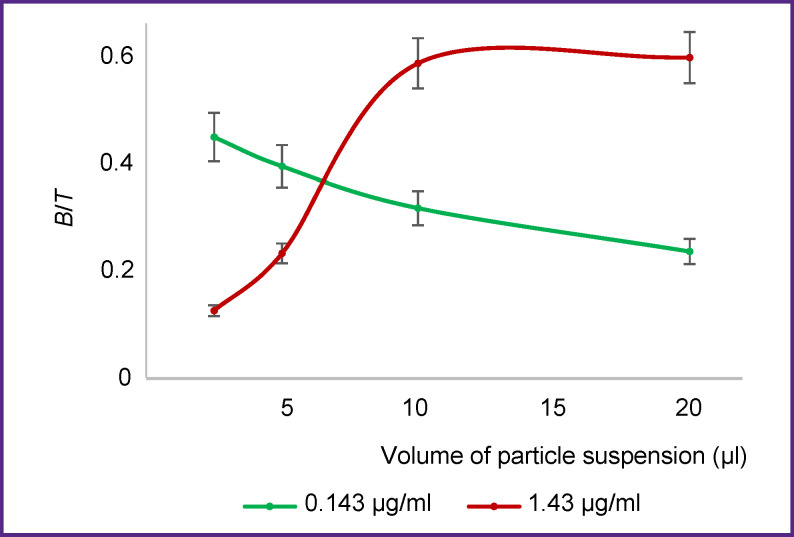
Dependence of *B*/*T* value on the number of magnetic particles in the sample Concentration of ^68^Ga-VHH-PD-L1В radioimmunoconjugate is indicated in the legend

We have tested the last statement by the experiment in which the radioimmunoconjugate was incubated with various numbers of particles in the samples (Figure 3). Suspensions of particles with immobilized antigen were added to the samples in the volume of 2.5, 5, 10, and 20 μl. Similarly, a series of samples was prepared with the same number of particles without antigen for determination of nonspecific binding. TBST with 1% BSA was added to the samples to obtain the total volume of 20 μl in a sample. Then, 50 μl of the solution containing ^68^Ga-VHH-PD-L1 at the concentration of 1.43 or 0.143 μg/ml was added to all samples. The experiment was further carried out as described in the Materials and Methods section.

When radioimmunoconjugate taken at the concentration of 1.43 μg/ml is binding to various numbers of antigen-coated magnetic particles, the saturation diagram reaches the plateau when antigen molecule excess is reached in the sample relative to the labeled VHH molecules (see Figure 3). However, if the concentration of radioimmunoconjugate is 10 times lower (0.143 μg/ml), the value of *B*/*T* ratio decreases with the increase of the particle content in the sample. It happens despite the obviously excessive amount of molecules of antigen immobilized on the particles in relation to the labeled VHH molecules. The results obtained confirm the previous conclusion stating that a certain ratio of radioimmunoconjugate concentrations and magnetic antigen-bearing particles is needed to measure IRF. Consequently, the estimation of radioimmunoconjugate IRF requires preparation of a sufficient number of samples with different concentrations of the labeled antigens for finding the maximum value of *B*/*T* ratio. Sharma et al. [10] performed a single-point analysis for one concentration of the labeled antibodies with a large excess of antigen molecules immobilized on magnetic particles to define IRF of the radioimmunoconjugates. The data presented in our work indicate that this approach does not ensure adequate results of the analysis. In other publications [13-15], the labeled antibodies of one concentration were incubated with various numbers of antigen-coated magnetic particles. However, as it is seen in Figure 3, this approach may also give erroneous estimation of IRF, as it is difficult to use a sufficiently wide range of concentrations of such particles due to the experiment conditions.

**Figure 4.**
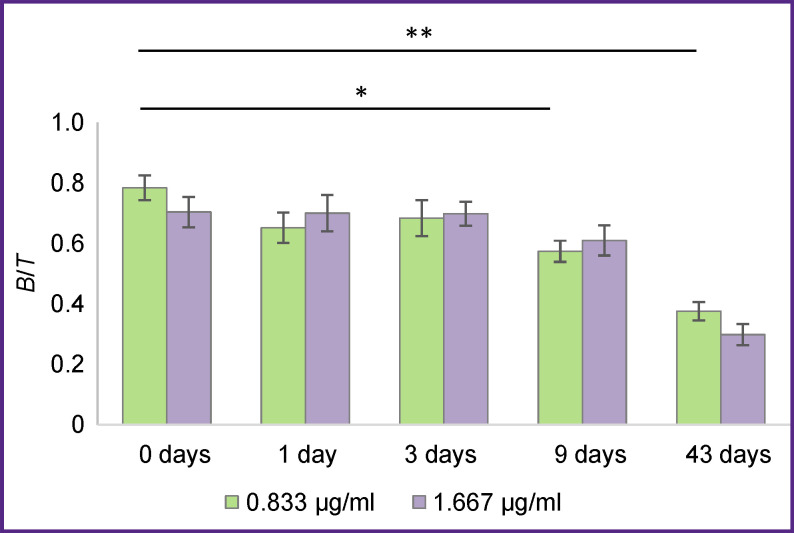
*B*/*T* ratio measured 0, 1, 3, 9 and 43 days after immobilization of antigen molecules on magnetic particles Concentration of ^68^Ga-VHH-PD-L1 radioimmunoconjugate is indicated in the legend; * p<0.05; ** p<0.01

Magnetic particles precoated with antigen molecules were used to determine IRF of the ^68^Ga-VHH-PD-L1 radioimmunoconjugate. The shelf-life testing was performed for the particles with immobilized antigen stored at 4–8°C (Figure 4).

Data presented in Figure 4 show that storage of the particles with immobilized antigen molecules for 3 days does not reduce the measured IRF value of the ^68^Ga-VHH-PD-L1 radioimmunoconjugate. This value drops from 78.5 to 61.1% if particles are stored for 9 days, and to 37.6% after a 43-day storage. Thus, after immobilization of antigen molecules, magnetic particles remain suitable for measuring IRF at least for 3 days.

**Figure 5.**
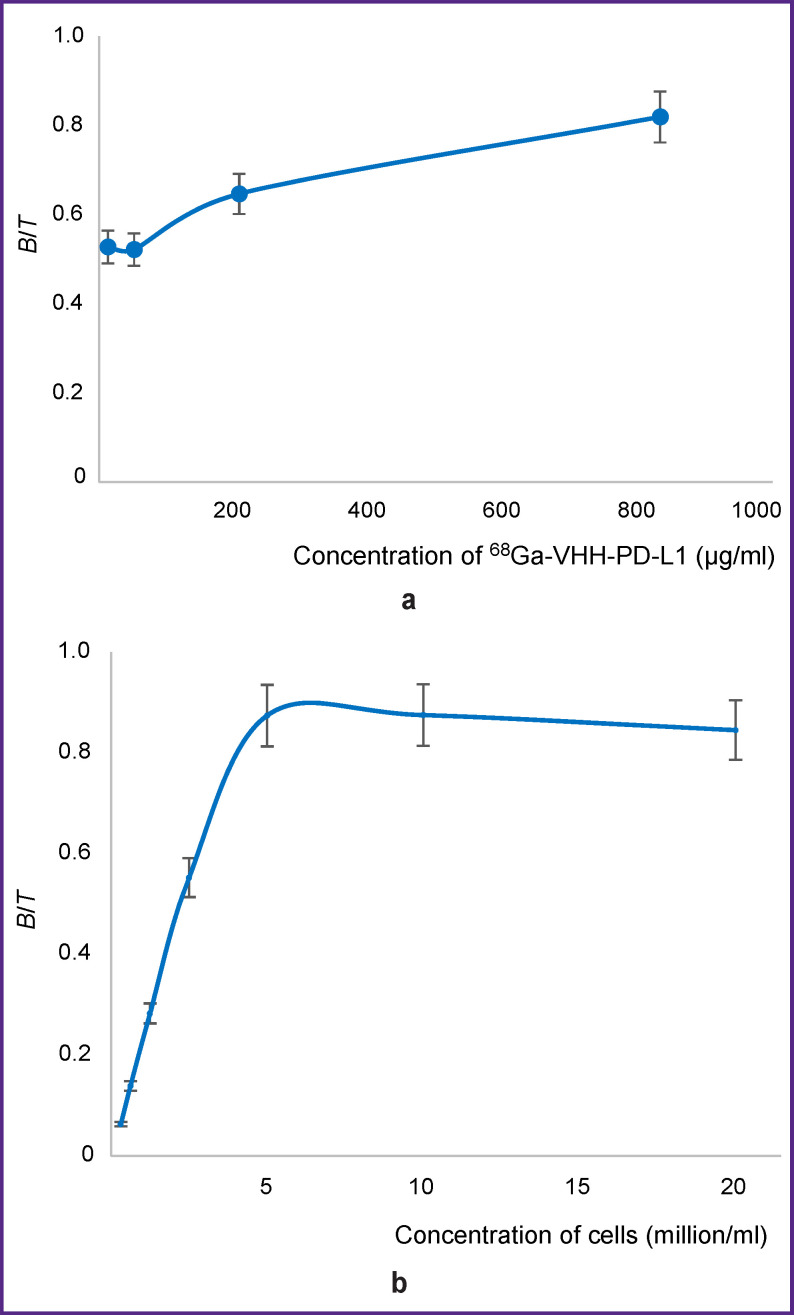
Determination of the ^68^Ga-VHH-PD-L1 IRF value by two methods: (a) by incubation of radioimmunoconjugate with magnetic particles; (b) by incubation of radioimmunoconjugate with CT26-PD-L1 cells

In our study, we compared the IRF values of one ^68^Ga-VHH-PD-L1 radioimmunoconjugate sample measured by the application of magnetic particles and the cells expressing the same antigen on their surface (Figure 5). This investigation was of great importance since immobilization of the protein antigen on the particles may change its conformation and influence binding to antibodies [26].

The mean IRF values determined by binding of radioimmunoconjugate to magnetic particles and CT26-PD-L cells were 81.2 and 87.5%, respectively (see Figure 5). These values are comparable, which speaks of the adequacy of using the method presented in this paper for the evaluation of the specific activity of radioimmunoconjugates. The radioimmunoconjugate preserves the affinity to the PD-L1 molecules immobilized on the particles despite the fact that their conformation may differ from the native conformation of PD-L1 molecules expressed on the cell surface. Steric accessibility for the sites of binding of immobilized antigen to radioimmunoconjugate is also preserved.

**Figure 6.**
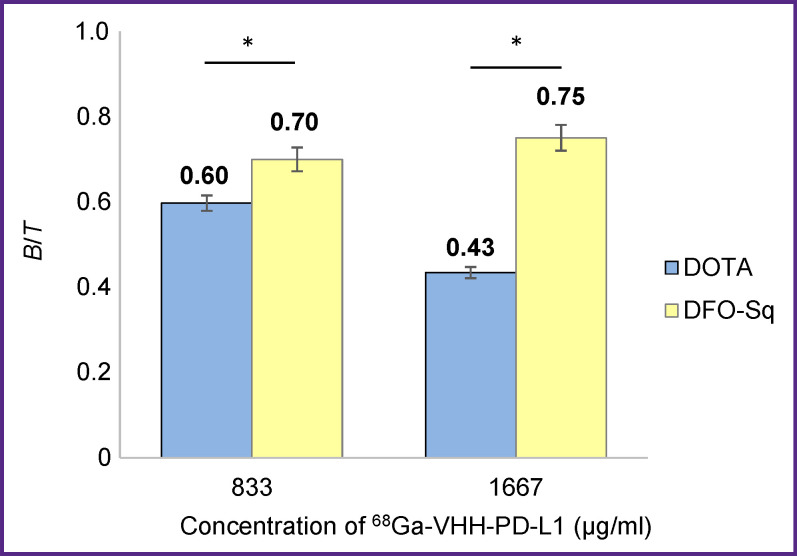
Comparison of *B*/*T* values measured for ^68^Ga-VHH-PD-L1 radioimmunoconjugates labeled using chelating precursors DOTA-VHH-PD-L1 and FO-Sq-VHH-PD-L1 * p<0.05

We used magnetic particles for comparative investigations of radioimmunoconjugates prepared with the help of various methods. DOTA-VHH-PD-L1 and DFO-Sq-VHH-PD-L1 were employed as chelating precursors for labeling with ^68^Ga radionuclide (Figure 6). According to the experimental data, the IRF value of ^68^Ga-VHH-PD-L1 containing DFO-Sq is 75.1%, whereas a similar radioimmunoconjugate obtained by labeling VHH modified with the DOTA chelator had the IRF value of 59.7%. It means that usage of DFO-Sq instead of DOTA allows for the increase of the undamaged portion of the labeled VHHs.

**Figure 7.**
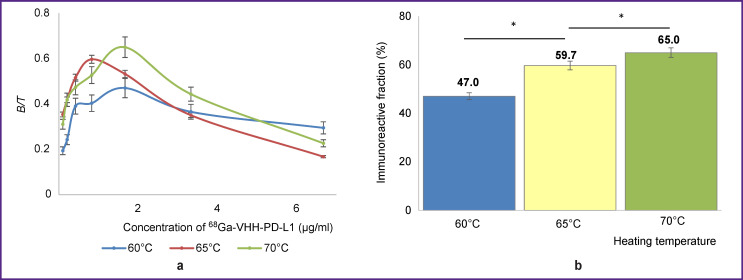
The effect of reaction mixture temperature on the IRF value of the ^68^Ga-VHH-PD-L1 radioimmunoconjugate containing DOTA chelator: (a) dependence of *B*/*T* value on the concentration of radioimmunoconjugate; (b) value of the measured IRF of radioimmunoconjugate obtained at different temperatures of the reaction mixture; * p<0.05

Based on the results of the experiment, the task was set to find the conditions of labeling VHH antibodies with ^68^Ga radionuclide using a DOTA chelator in order to increase IRF of the radiopharmaceutical. For labeling VHH antibodies with ^68^Ga radionuclide, the reaction mixture was heated to 60, 65, or 70°C. Then, IRF of the obtained ^68^Ga-VHH-PD-L1 radioimmunoconjugate was determined using magnetic particles (Figure 7).

The data of Figure 7 show that elevation of the heating temperature in the process of radioimmunoconjugate production promoted the increase of the IRF value. Thus, heating to 60°C gave IRF value of 47.0%, heating to 65°C — 59.7%, while heating to 70°C increased the value to 65.0%. It is obvious that heating the reaction mixture to 70°C results in the production of the radioimmunoconjugate with a higher IRF value than heating at lower temperatures. The reasons for this effect are not yet clear.

The above-mentioned results show that application of magnetic particles with antigen molecules immobilized on their surface allow one to determine optimal conditions for ^68^Ga radionuclide labeling of VHHs against PD-L1.

The present study has demonstrated that in assessing specific activity of ^68^Ga-VHH-PD-L1 radioimmunoconjugate, magnetic particles coated with PD-L1 antigen are able to replace the cells expressing PD-L1 on their surface. In the future, it is necessary to study the possibility to apply the presented method for determining IRF of other radioimmunoconjugates based on VHH antibodies and other antigen-binding molecules. There are two evident directions of work to improve the method: to increase the shelf-life of the antigen-coated magnetic particles from three to at least seven days, and to widen the range of radioimmunoconjugate concentrations in a sample suitable for making measurements.

## Conclusion

Application of magnetic particles coated with immobilized antigen molecules provides the possibility to determine immunoreactive fraction of VHH antibodies against ^68^Ga-labeled PD-L1 for 15 min. The obtained results show that this method has a real practical value for measuring specific activity of radioimmunoconjugates *in vitro* since the analysis procedure becomes more available and reliable than in case of using cells. Of special importance is the fact that the application of magnetic particles opens up the possibility of a fast routine assessment of radiopharmaceuticals containing short-lived isotopes.
